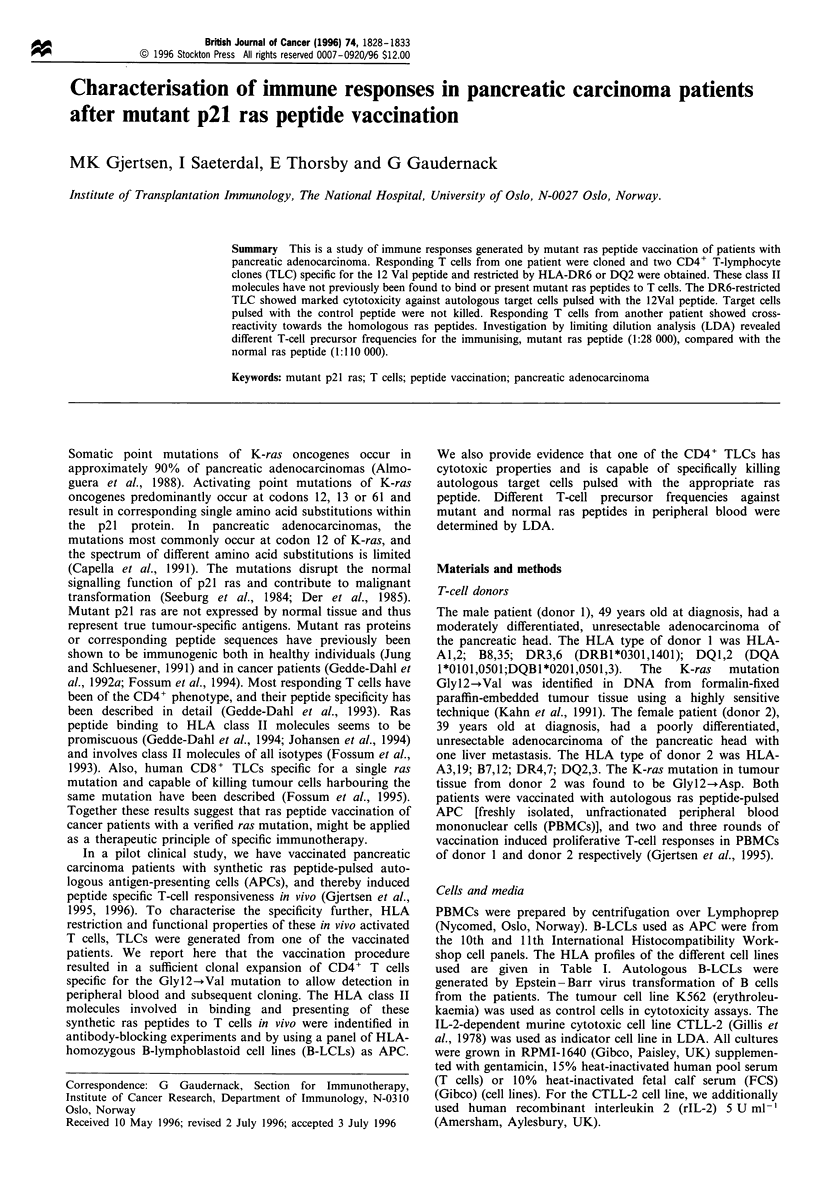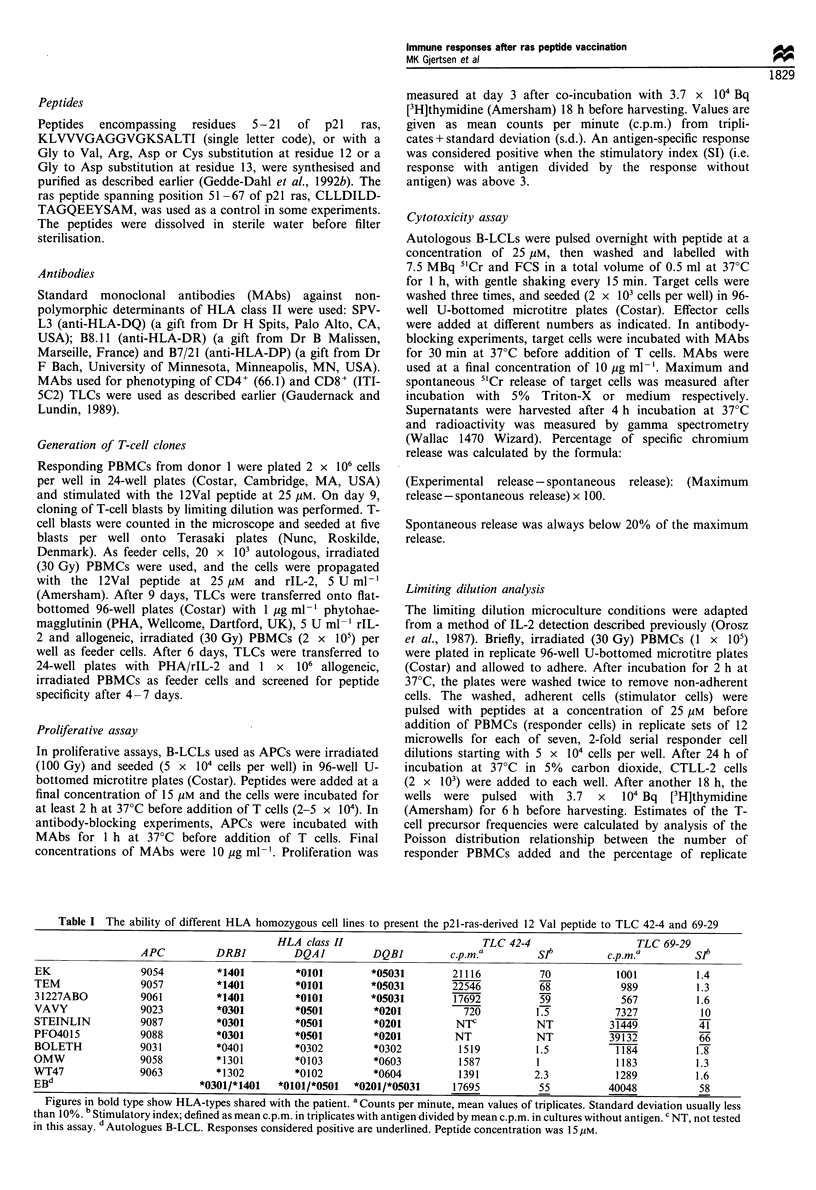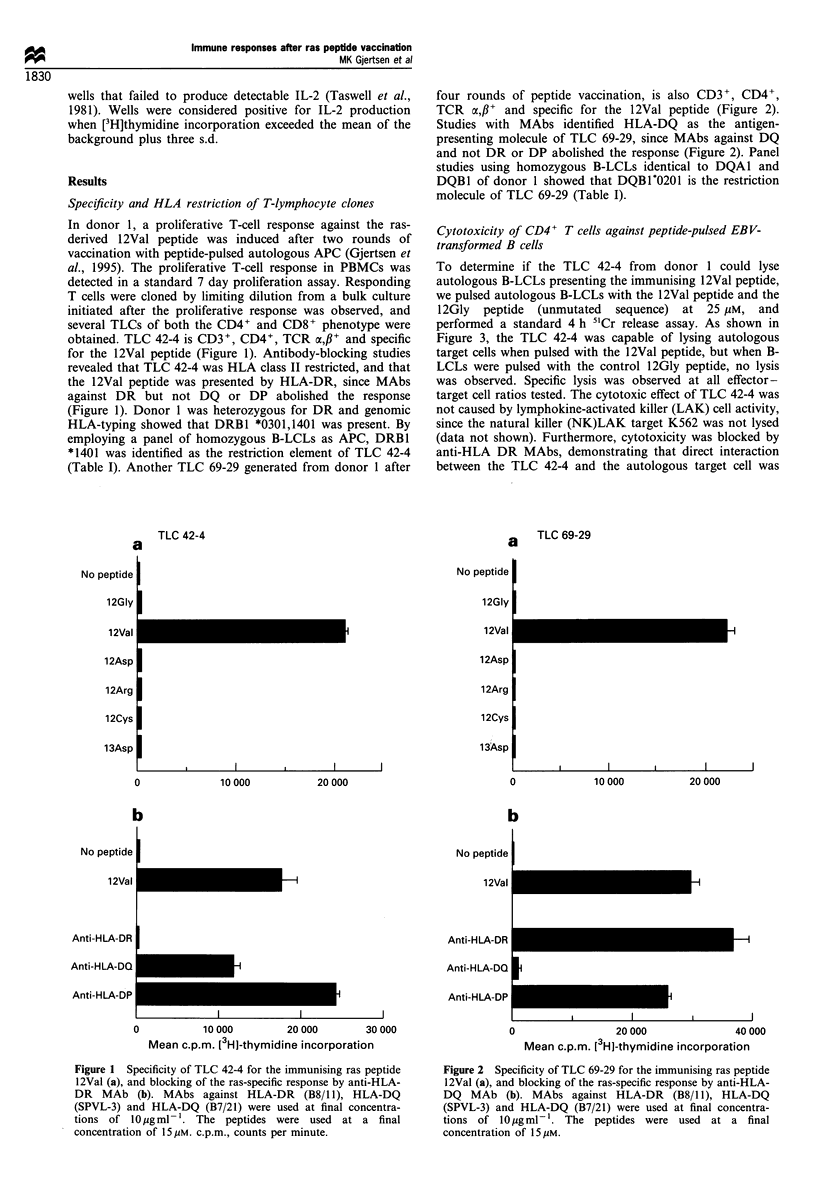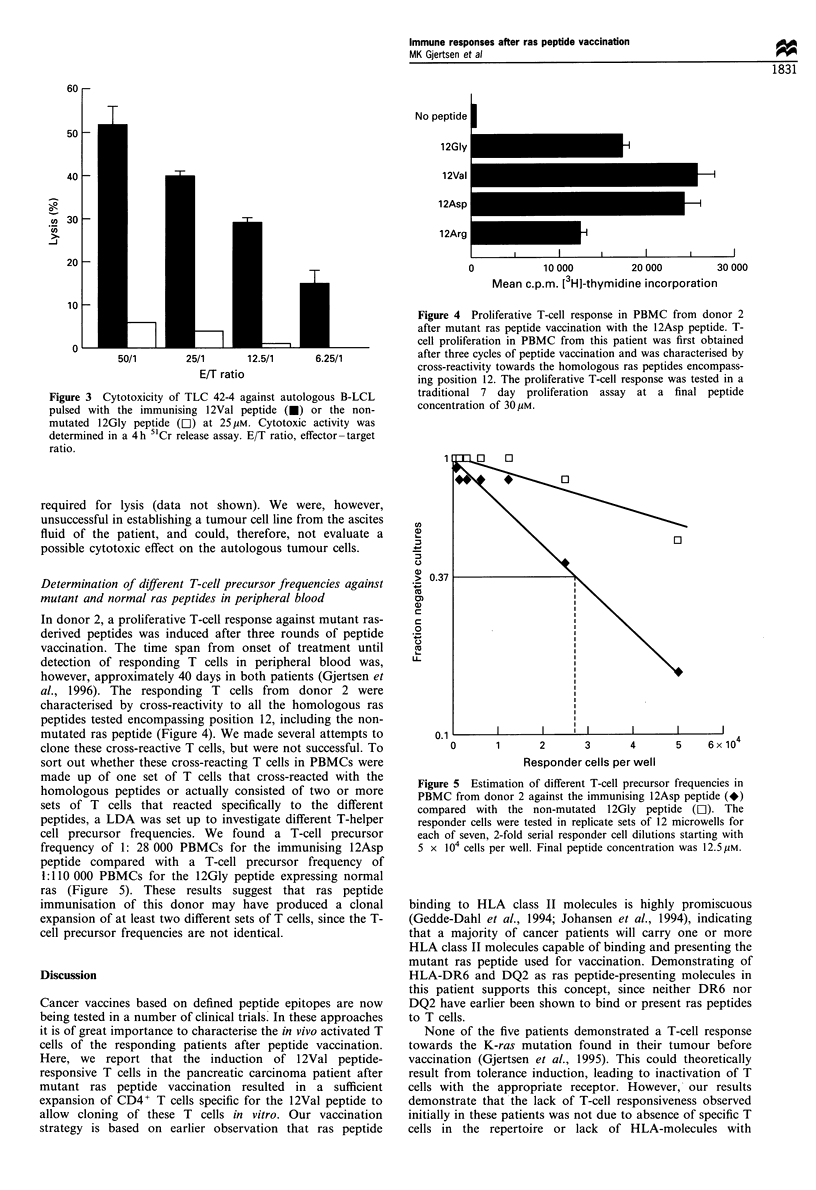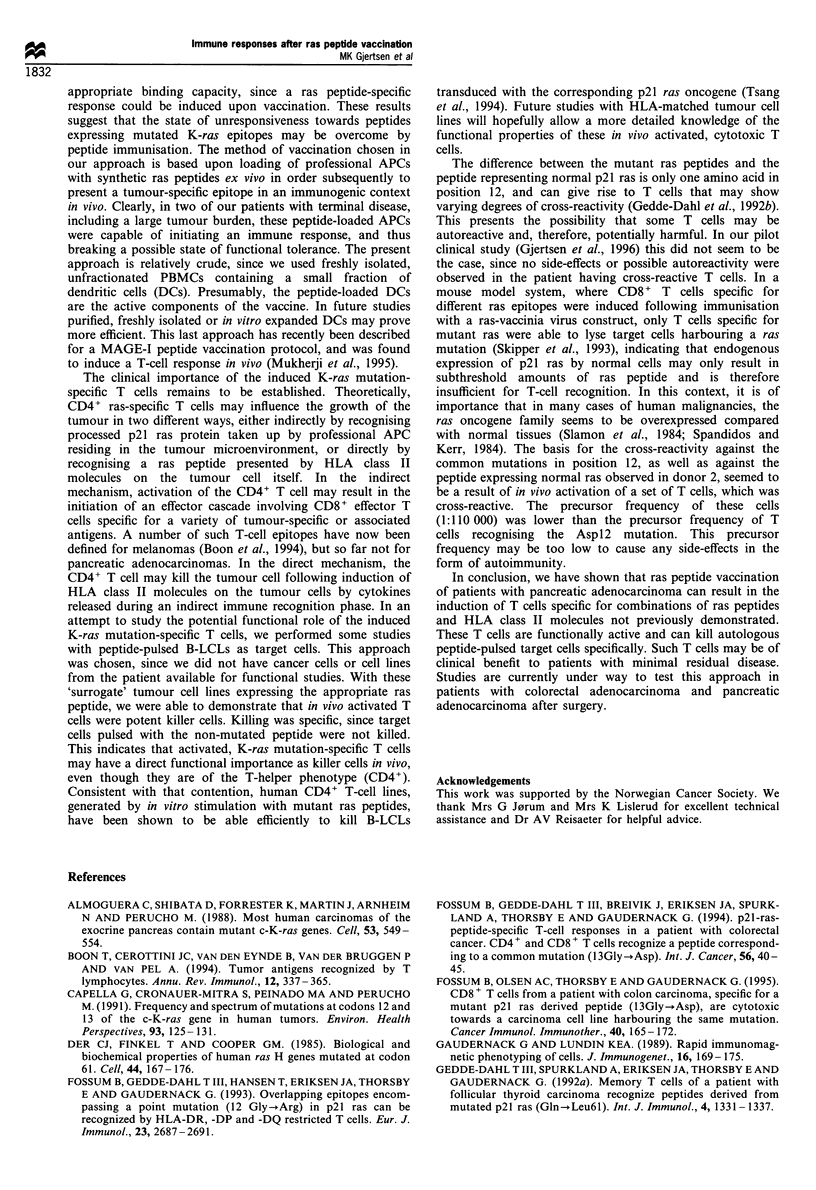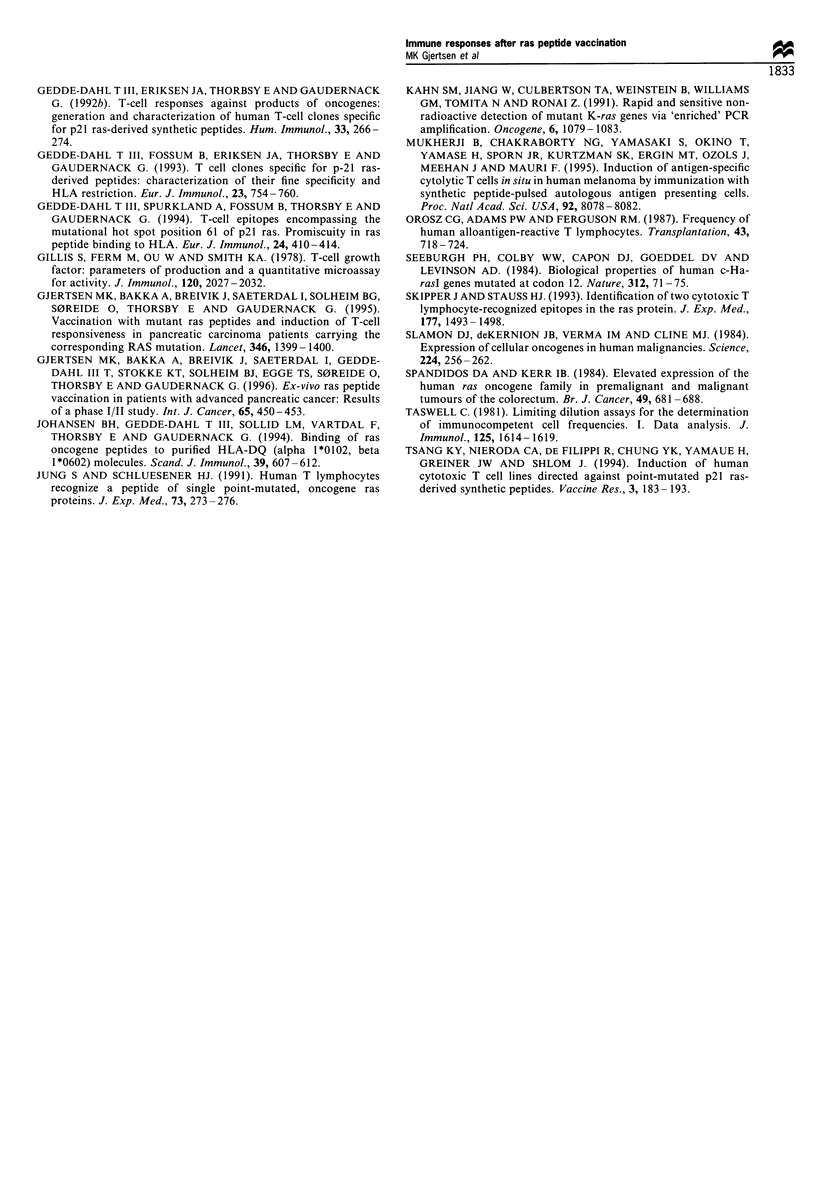# Characterisation of immune responses in pancreatic carcinoma patients after mutant p21 ras peptide vaccination.

**DOI:** 10.1038/bjc.1996.638

**Published:** 1996-12

**Authors:** M. K. Gjertsen, I. Saeterdal, E. Thorsby, G. Gaudernack

**Affiliations:** Institute of Transplantation Immunology, National Hospital, University of Oslo, Norway.

## Abstract

This is a study of immune responses generated by mutant ras peptide vaccination of patients with pancreatic adenocarcinoma. Responding T cells from one patient were cloned and two CD4+ T-lymphocyte clones (TLC) specific for the 12 Val peptide and restricted by HLA-DR6 or DQ2 were obtained. These class II molecules have not previously been found to bind or present mutant ras peptides to T cells. The DR6-restricted TLC showed marked cytotoxicity against autologous target cells pulsed with the 12 Val peptide. Target cells pulsed with the control peptide were not killed. Responding T cells from another patient showed cross-reactivity towards the homologous ras peptides. Investigation by limiting dilution analysis (LDA) revealed different T-cell precursor frequencies for the immunising, mutant ras peptide (1:28000), compared with the normal ras peptide (1:110000).


British Journal of Cancer (1996) 74, 1828-1833
© 1996 Stockton Press All rights reserved 0007-0920/96 $12.00

### Characterisation of immune responses in pancreatic carcinoma patients after mutant p21 ras peptide vaccination

MK Gjertsen, I Saeterdal, E Thorsby and G Gaudernack

*Institute of Transplantation Immunology, The National Hospital, University of Oslo, N-0027 Oslo, Norway.*

**Summary** This is a study of immune responses generated by mutant ras peptide vaccination of patients with pancreatic adenocarcinoma. Responding T cells from one patient were cloned and two $CD4^+$ T-lymphocyte clones (TLC) specific for the 12 Val peptide and restricted by HLA-DR6 or DQ2 were obtained. These class II molecules have not previously been found to bind or present mutant ras peptides to T cells. The DR6-restricted TLC showed marked cytotoxicity against autologous target cells pulsed with the 12Val peptide. Target cells pulsed with the control peptide were not killed. Responding T cells from another patient showed cross-reactivity towards the homologous ras peptides. Investigation by limiting dilution analysis (LDA) revealed different T-cell precursor frequencies for the immunising, mutant ras peptide (1:28 000), compared with the normal ras peptide (1:110 000).

**Keywords:** mutant p21 ras; T cells; peptide vaccination; pancreatic adenocarcinoma

Somatic point mutations of K-*ras* oncogenes occur in approximately 90% of pancreatic adenocarcinomas (Almoguera *et al.*, 1988). Activating point mutations of K-*ras* oncogenes predominantly occur at codons 12, 13 or 61 and result in corresponding single amino acid substitutions within the p21 protein. In pancreatic adenocarcinomas, the mutations most commonly occur at codon 12 of K-*ras*, and the spectrum of different amino acid substitutions is limited (Capella *et al.*, 1991). The mutations disrupt the normal signalling function of p21 ras and contribute to malignant transformation (Seeburg *et al.*, 1984; Der *et al.*, 1985). Mutant p21 ras are not expressed by normal tissue and thus represent true tumour-specific antigens. Mutant ras proteins or corresponding peptide sequences have previously been shown to be immunogenic both in healthy individuals (Jung and Schluesener, 1991) and in cancer patients (Gedde-Dahl *et al.*, 1992*a*; Fossum *et al.*, 1994). Most responding T cells have been of the $CD4^+$ phenotype, and their peptide specificity has been described in detail (Gedde-Dahl *et al.*, 1993). Ras peptide binding to HLA class II molecules seems to be promiscuous (Gedde-Dahl *et al.*, 1994; Johansen *et al.*, 1994) and involves class II molecules of all isotypes (Fossum *et al.*, 1993). Also, human $CD8^+$ TLCs specific for a single *ras* mutation and capable of killing tumour cells harbouring the same mutation have been described (Fossum *et al.*, 1995). Together these results suggest that ras peptide vaccination of cancer patients with a verified *ras* mutation, might be applied as a therapeutic principle of specific immunotherapy.

In a pilot clinical study, we have vaccinated pancreatic carcinoma patients with synthetic ras peptide-pulsed autologous antigen-presenting cells (APCs), and thereby induced peptide specific T-cell responsiveness *in vivo* (Gjertsen *et al.*, 1995, 1996). To characterise the specificity further, HLA restriction and functional properties of these *in vivo* activated T cells, TLCs were generated from one of the vaccinated patients. We report here that the vaccination procedure resulted in a sufficient clonal expansion of $CD4^+$ T cells specific for the Gly12→Val mutation to allow detection in peripheral blood and subsequent cloning. The HLA class II molecules involved in binding and presenting of these synthetic ras peptides to T cells *in vivo* were indentified in antibody-blocking experiments and by using a panel of HLA-homozygous B-lymphoblastoid cell lines (B-LCLs) as APC. We also provide evidence that one of the $CD4^+$ TLCs has cytotoxic properties and is capable of specifically killing autologous target cells pulsed with the appropriate ras peptide. Different T-cell precursor frequencies against mutant and normal ras peptides in peripheral blood were determined by LDA.

Correspondence: G Gaudernack, Section for Immunotherapy, Institute of Cancer Research, Department of Immunology, N-0310 Oslo, Norway
Received 10 May 1996; revised 2 July 1996; accepted 3 July 1996

### Materials and methods

### *T-cell donors*

The male patient (donor 1), 49 years old at diagnosis, had a moderately differentiated, unresectable adenocarcinoma of the pancreatic head. The HLA type of donor 1 was HLA-A1,2; B8,35; DR3,6 (DRB1*0301,1401); DQ1,2 (DQA 1*0101,0501;DQB1*0201,0501,3). The K-*ras* mutation Gly12→Val was identified in DNA from formalin-fixed paraffin-embedded tumour tissue using a highly sensitive technique (Kahn *et al.*, 1991). The female patient (donor 2), 39 years old at diagnosis, had a poorly differentiated, unresectable adenocarcinoma of the pancreatic head with one liver metastasis. The HLA type of donor 2 was HLA-A3,19; B7,12; DR4,7; DQ2,3. The K-*ras* mutation in tumour tissue from donor 2 was found to be Gly12→Asp. Both patients were vaccinated with autologous ras peptide-pulsed APC [freshly isolated, unfractionated peripheral blood mononuclear cells (PBMCs)], and two and three rounds of vaccination induced proliferative T-cell responses in PBMCs of donor 1 and donor 2 respectively (Gjertsen *et al.*, 1995).

### *Cells and media*

PBMCs were prepared by centrifugation over Lymphoprep (Nycomed, Oslo, Norway). B-LCLs used as APC were from the 10th and 11th International Histocompatibility Workshop cell panels. The HLA profiles of the different cell lines used are given in Table I. Autologous B-LCLs were generated by Epstein–Barr virus transformation of B cells from the patients. The tumour cell line K562 (erythroleukaemia) was used as control cells in cytotoxicity assays. The IL-2-dependent murine cytotoxic cell line CTLL-2 (Gillis *et al.*, 1978) was used as indicator cell line in LDA. All cultures were grown in RPMI-1640 (Gibco, Paisley, UK) supplemented with gentamicin, 15% heat-inactivated human pool serum (T cells) or 10% heat-inactivated fetal calf serum (FCS) (Gibco) (cell lines). For the CTLL-2 cell line, we additionally used human recombinant interleukin 2 (rIL-2) 5 U $ml^{-1}$ (Amersham, Aylesbury, UK).

*Peptides*

Peptides encompassing residues 5–21 of p21 ras, KLVVVGAGGVGKSALTI (single letter code), or with a Gly to Val, Arg, Asp or Cys substitution at residue 12 or a Gly to Asp substitution at residue 13, were synthesised and purified as described earlier (Gedde-Dahl *et al.*, 1992*b*). The ras peptide spanning position 51–67 of p21 ras, CLLDILDTAGQEEYSAM, was used as a control in some experiments. The peptides were dissolved in sterile water before filter sterilisation.

*Antibodies*

Standard monoclonal antibodies (MAbs) against non-polymorphic determinants of HLA class II were used: SPV-L3 (anti-HLA-DQ) (a gift from Dr H Spits, Palo Alto, CA, USA); B8.11 (anti-HLA-DR) (a gift from Dr B Malissen, Marseille, France) and B7/21 (anti-HLA-DP) (a gift from Dr F Bach, University of Minnesota, Minneapolis, MN, USA). MAbs used for phenotyping of $CD4^+$ (66.1) and $CD8^+$ (ITI-5C2) TLCs were used as described earlier (Gaudernack and Lundin, 1989).

*Generation of T-cell clones*

Responding PBMCs from donor 1 were plated $2 \times 10^6$ cells per well in 24-well plates (Costar, Cambridge, MA, USA) and stimulated with the 12Val peptide at 25 $\mu$M. On day 9, cloning of T-cell blasts by limiting dilution was performed. T-cell blasts were counted in the microscope and seeded at five blasts per well onto Terasaki plates (Nunc, Roskilde, Denmark). As feeder cells, $20 \times 10^3$ autologous, irradiated (30 Gy) PBMCs were used, and the cells were propagated with the 12Val peptide at 25 $\mu$M and rIL-2, 5 U $ml^{-1}$ (Amersham). After 9 days, TLCs were transferred onto flat-bottomed 96-well plates (Costar) with 1 $\mu g\ ml^{-1}$ phytohaemagglutinin (PHA, Wellcome, Dartford, UK), 5 U $ml^{-1}$ rIL-2 and allogeneic, irradiated (30 Gy) PBMCs ($2 \times 10^5$) per well as feeder cells. After 6 days, TLCs were transferred to 24-well plates with PHA/rIL-2 and $1 \times 10^6$ allogeneic, irradiated PBMCs as feeder cells and screened for peptide specificity after 4–7 days.

*Proliferative assay*

In proliferative assays, B-LCLs used as APCs were irradiated (100 Gy) and seeded ($5 \times 10^4$ cells per well) in 96-well U-bottomed microtitre plates (Costar). Peptides were added at a final concentration of 15 $\mu$M and the cells were incubated for at least 2 h at 37°C before addition of T cells (2-5 $\times 10^4$). In antibody-blocking experiments, APCs were incubated with MAbs for 1 h at 37°C before addition of T cells. Final concentrations of MAbs were 10 $\mu g\ ml^{-1}$. Proliferation was measured at day 3 after co-incubation with $3.7 \times 10^4$ Bq [$^3$H]thymidine (Amersham) 18 h before harvesting. Values are given as mean counts per minute (c.p.m.) from triplicates + standard deviation (s.d.). An antigen-specific response was considered positive when the stimulatory index (SI) (i.e. response with antigen divided by the response without antigen) was above 3.

*Cytotoxicity assay*

Autologous B-LCLs were pulsed overnight with peptide at a concentration of 25 $\mu$M, then washed and labelled with 7.5 MBq $^{51}$Cr and FCS in a total volume of 0.5 ml at 37°C for 1 h, with gentle shaking every 15 min. Target cells were washed three times, and seeded ($2 \times 10^3$ cells per well) in 96-well U-bottomed microtitre plates (Costar). Effector cells were added at different numbers as indicated. In antibody-blocking experiments, target cells were incubated with MAbs for 30 min at 37°C before addition of T cells. MAbs were used at a final concentration of 10 $\mu g\ ml^{-1}$. Maximum and spontaneous $^{51}$Cr release of target cells was measured after incubation with 5% Triton-X or medium respectively. Supernatants were harvested after 4 h incubation at 37°C and radioactivity was measured by gamma spectrometry (Wallac 1470 Wizard). Percentage of specific chromium release was calculated by the formula:

(Experimental release – spontaneous release): (Maximum release – spontaneous release) × 100.

Spontaneous release was always below 20% of the maximum release.

*Limiting dilution analysis*

The limiting dilution microculture conditions were adapted from a method of IL-2 detection described previously (Orosz *et al.*, 1987). Briefly, irradiated (30 Gy) PBMCs ($1 \times 10^5$) were plated in replicate 96-well U-bottomed microtitre plates (Costar) and allowed to adhere. After incubation for 2 h at 37°C, the plates were washed twice to remove non-adherent cells. The washed, adherent cells (stimulator cells) were pulsed with peptides at a concentration of 25 $\mu$M before addition of PBMCs (responder cells) in replicate sets of 12 microwells for each of seven, 2-fold serial responder cell dilutions starting with $5 \times 10^4$ cells per well. After 24 h of incubation at 37°C in 5% carbon dioxide, CTLL-2 cells ($2 \times 10^3$) were added to each well. After another 18 h, the wells were pulsed with $3.7 \times 10^4$ Bq [$^3$H]thymidine (Amersham) for 6 h before harvesting. Estimates of the T-cell precursor frequencies were calculated by analysis of the Poisson distribution relationship between the number of responder PBMCs added and the percentage of replicate

**Table I** The ability of different HLA homozygous cell lines to present the p21-ras-derived 12 Val peptide to TLC 42-4 and 69-29

| | | HLA class II | | | TLC 42-4 | | TLC 69-29 | |
|---|---|---|---|---|---|---|---|---|
| | *APC* | *DRB1* | *DQA1* | *DQB1* | *c.p.m.*[a] | *SI*[b] | *c.p.m.*[a] | *SI*[b] |
| EK | 9054 | ***1401** | ***0101** | ***05031** | 21116 | 70 | 1001 | 1.4 |
| TEM | 9057 | ***1401** | ***0101** | ***05031** | 22546 | 68 | 989 | 1.3 |
| 31227ABO | 9061 | ***1401** | ***0101** | ***05031** | 17692 | 59 | 567 | 1.6 |
| VAVY | 9023 | ***0301** | ***0501** | ***0201** | 720 | 1.5 | 7327 | 10 |
| STEINLIN | 9087 | ***0301** | ***0501** | ***0201** | NT[c] | NT | 31449 | 41 |
| PFO4015 | 9088 | ***0301** | ***0501** | ***0201** | NT | NT | 39132 | 66 |
| BOLETH | 9031 | *0401 | *0302 | *0302 | 1519 | 1.5 | 1184 | 1.8 |
| OMW | 9058 | *1301 | *0103 | *0603 | 1587 | 1 | 1183 | 1.3 |
| WT47 | 9063 | *1302 | *0102 | *0604 | 1391 | 2.3 | 1289 | 1.6 |
| EB[d] | | ***0301/*1401** | ***0101/*0501** | ***0201/*05031** | 17695 | 55 | 40048 | 58 |

Figures in bold type show HLA-types shared with the patient. [a] Counts per minute, mean values of triplicates. Standard deviation usually less than 10%. [b] Stimulatory index; defined as mean c.p.m. in triplicates with antigen divided by mean c.p.m. in cultures without antigen. [c] NT, not tested in this assay. [d] Autologues B-LCL. Responses considered positive are underlined. Peptide concentration was 15 $\mu$M.

wells that failed to produce detectable IL-2 (Taswell *et al.*, 1981). Wells were considered positive for IL-2 production when [$^3$H]thymidine incorporation exceeded the mean of the background plus three s.d.

### Results

### *Specificity and HLA restriction of T-lymphocyte clones*

In donor 1, a proliferative T-cell response against the ras-derived 12Val peptide was induced after two rounds of vaccination with peptide-pulsed autologous APC (Gjertsen *et al.*, 1995). The proliferative T-cell response in PBMCs was detected in a standard 7 day proliferation assay. Responding T cells were cloned by limiting dilution from a bulk culture initiated after the proliferative response was observed, and several TLCs of both the CD4$^+$ and CD8$^+$ phenotype were obtained. TLC 42-4 is CD3$^+$, CD4$^+$, TCR $\alpha,\beta^+$ and specific for the 12Val peptide (Figure 1). Antibody-blocking studies revealed that TLC 42-4 was HLA class II restricted, and that the 12Val peptide was presented by HLA-DR, since MAbs against DR but not DQ or DP abolished the response (Figure 1). Donor 1 was heterozygous for DR and genomic HLA-typing showed that DRB1 *0301,1401 was present. By employing a panel of homozygous B-LCLs as APC, DRB1 *1401 was identified as the restriction element of TLC 42-4 (Table I). Another TLC 69-29 generated from donor 1 after four rounds of peptide vaccination, is also CD3$^+$, CD4$^+$, TCR $\alpha,\beta^+$ and specific for the 12Val peptide (Figure 2). Studies with MAbs identified HLA-DQ as the antigen-presenting molecule of TLC 69-29, since MAbs against DQ and not DR or DP abolished the response (Figure 2). Panel studies using homozygous B-LCLs identical to DQA1 and DQB1 of donor 1 showed that DQB1*0201 is the restriction molecule of TLC 69-29 (Table I).

### *Cytotoxicity of CD4$^+$ T cells against peptide-pulsed EBV-transformed B cells*

To determine if the TLC 42-4 from donor 1 could lyse autologous B-LCLs presenting the immunising 12Val peptide, we pulsed autologous B-LCLs with the 12Val peptide and the 12Gly peptide (unmutated sequence) at 25 $\mu$M, and performed a standard 4 h $^{51}$Cr release assay. As shown in Figure 3, the TLC 42-4 was capable of lysing autologous target cells when pulsed with the 12Val peptide, but when B-LCLs were pulsed with the control 12Gly peptide, no lysis was observed. Specific lysis was observed at all effector–target cell ratios tested. The cytotoxic effect of TLC 42-4 was not caused by lymphokine-activated killer (LAK) cell activity, since the natural killer (NK)LAK target K562 was not lysed (data not shown). Furthermore, cytotoxicity was blocked by anti-HLA DR MAbs, demonstrating that direct interaction between the TLC 42-4 and the autologous target cell was

**Figure 1** Specificity of TLC 42-4 for the immunising ras peptide 12Val (**a**), and blocking of the ras-specific response by anti-HLA-DR MAb (**b**). MAbs against HLA-DR (B8/11), HLA-DQ (SPVL-3) and HLA-DQ (B7/21) were used at final concentrations of 10 $\mu$g ml$^{-1}$. The peptides were used at a final concentration of 15 $\mu$M. c.p.m., counts per minute.

**Figure 2** Specificity of TLC 69-29 for the immunising ras peptide 12Val (**a**), and blocking of the ras-specific response by anti-HLA-DQ MAb (**b**). MAbs against HLA-DR (B8/11), HLA-DQ (SPVL-3) and HLA-DQ (B7/21) were used at final concentrations of 10 $\mu$g ml$^{-1}$. The peptides were used at a final concentration of 15 $\mu$M.

**Figure 3** Cytotoxicity of TLC 42-4 against autologous B-LCL pulsed with the immunising 12Val peptide (■) or the non-mutated 12Gly peptide (□) at 25 μM. Cytotoxic activity was determined in a 4 h $^{51}$Cr release assay. E/T ratio, effector–target ratio.

required for lysis (data not shown). We were, however, unsuccessful in establishing a tumour cell line from the ascites fluid of the patient, and could, therefore, not evaluate a possible cytotoxic effect on the autologous tumour cells.

*Determination of different T-cell precursor frequencies against mutant and normal ras peptides in peripheral blood*

In donor 2, a proliferative T-cell response against mutant ras-derived peptides was induced after three rounds of peptide vaccination. The time span from onset of treatment until detection of responding T cells in peripheral blood was, however, approximately 40 days in both patients (Gjertsen *et al.*, 1996). The responding T cells from donor 2 were characterised by cross-reactivity to all the homologous ras peptides tested encompassing position 12, including the non-mutated ras peptide (Figure 4). We made several attempts to clone these cross-reactive T cells, but were not successful. To sort out whether these cross-reacting T cells in PBMCs were made up of one set of T cells that cross-reacted with the homologous peptides or actually consisted of two or more sets of T cells that reacted specifically to the different peptides, a LDA was set up to investigate different T-helper cell precursor frequencies. We found a T-cell precursor frequency of 1: 28 000 PBMCs for the immunising 12Asp peptide compared with a T-cell precursor frequency of 1:110 000 PBMCs for the 12Gly peptide expressing normal ras (Figure 5). These results suggest that ras peptide immunisation of this donor may have produced a clonal expansion of at least two different sets of T cells, since the T-cell precursor frequencies are not identical.

### Discussion

Cancer vaccines based on defined peptide epitopes are now being tested in a number of clinical trials. In these approaches it is of great importance to characterise the *in vivo* activated T cells of the responding patients after peptide vaccination. Here, we report that the induction of 12Val peptide-responsive T cells in the pancreatic carcinoma patient after mutant ras peptide vaccination resulted in a sufficient expansion of CD4$^+$ T cells specific for the 12Val peptide to allow cloning of these T cells *in vitro*. Our vaccination strategy is based on earlier observation that ras peptide

**Figure 4** Proliferative T-cell response in PBMC from donor 2 after mutant ras peptide vaccination with the 12Asp peptide. T-cell proliferation in PBMC from this patient was first obtained after three cycles of peptide vaccination and was characterised by cross-reactivity towards the homologous ras peptides encompassing position 12. The proliferative T-cell response was tested in a traditional 7 day proliferation assay at a final peptide concentration of 30 μM.

**Figure 5** Estimation of different T-cell precursor frequencies in PBMC from donor 2 against the immunising 12Asp peptide (◆) compared with the non-mutated 12Gly peptide (□). The responder cells were tested in replicate sets of 12 microwells for each of seven, 2-fold serial responder cell dilutions starting with 5 × 10$^4$ cells per well. Final peptide concentration was 12.5 μM.

binding to HLA class II molecules is highly promiscuous (Gedde-Dahl *et al.*, 1994; Johansen *et al.*, 1994), indicating that a majority of cancer patients will carry one or more HLA class II molecules capable of binding and presenting the mutant ras peptide used for vaccination. Demonstrating of HLA-DR6 and DQ2 as ras peptide-presenting molecules in this patient supports this concept, since neither DR6 nor DQ2 have earlier been shown to bind or present ras peptides to T cells.

None of the five patients demonstrated a T-cell response towards the K-*ras* mutation found in their tumour before vaccination (Gjertsen *et al.*, 1995). This could theoretically result from tolerance induction, leading to inactivation of T cells with the appropriate receptor. However, our results demonstrate that the lack of T-cell responsiveness observed initially in these patients was not due to absence of specific T cells in the repertoire or lack of HLA-molecules with appropriate binding capacity, since a ras peptide-specific response could be induced upon vaccination. These results suggest that the state of unresponsiveness towards peptides expressing mutated K-*ras* epitopes may be overcome by peptide immunisation. The method of vaccination chosen in our approach is based upon loading of professional APCs with synthetic ras peptides *ex vivo* in order subsequently to present a tumour-specific epitope in an immunogenic context *in vivo*. Clearly, in two of our patients with terminal disease, including a large tumour burden, these peptide-loaded APCs were capable of initiating an immune response, and thus breaking a possible state of functional tolerance. The present approach is relatively crude, since we used freshly isolated, unfractionated PBMCs containing a small fraction of dendritic cells (DCs). Presumably, the peptide-loaded DCs are the active components of the vaccine. In future studies purified, freshly isolated or *in vitro* expanded DCs may prove more efficient. This last approach has recently been described for a MAGE-I peptide vaccination protocol, and was found to induce a T-cell response *in vivo* (Mukherji *et al.*, 1995).

The clinical importance of the induced K-*ras* mutation-specific T cells remains to be established. Theoretically, $CD4^+$ ras-specific T cells may influence the growth of the tumour in two different ways, either indirectly by recognising processed p21 ras protein taken up by professional APC residing in the tumour microenvironment, or directly by recognising a ras peptide presented by HLA class II molecules on the tumour cell itself. In the indirect mechanism, activation of the $CD4^+$ T cell may result in the initiation of an effector cascade involving $CD8^+$ effector T cells specific for a variety of tumour-specific or associated antigens. A number of such T-cell epitopes have now been defined for melanomas (Boon *et al.*, 1994), but so far not for pancreatic adenocarcinomas. In the direct mechanism, the $CD4^+$ T cell may kill the tumour cell following induction of HLA class II molecules on the tumour cells by cytokines released during an indirect immune recognition phase. In an attempt to study the potential functional role of the induced K-*ras* mutation-specific T cells, we performed some studies with peptide-pulsed B-LCLs as target cells. This approach was chosen, since we did not have cancer cells or cell lines from the patient available for functional studies. With these 'surrogate' tumour cell lines expressing the appropriate ras peptide, we were able to demonstrate that *in vivo* activated T cells were potent killer cells. Killing was specific, since target cells pulsed with the non-mutated peptide were not killed. This indicates that activated, K-*ras* mutation-specific T cells may have a direct functional importance as killer cells *in vivo*, even though they are of the T-helper phenotype ($CD4^+$). Consistent with that contention, human $CD4^+$ T-cell lines, generated by *in vitro* stimulation with mutant ras peptides, have been shown to be able efficiently to kill B-LCLs transduced with the corresponding p21 *ras* oncogene (Tsang *et al.*, 1994). Future studies with HLA-matched tumour cell lines will hopefully allow a more detailed knowledge of the functional properties of these *in vivo* activated, cytotoxic T cells.

The difference between the mutant ras peptides and the peptide representing normal p21 ras is only one amino acid in position 12, and can give rise to T cells that may show varying degrees of cross-reactivity (Gedde-Dahl *et al.*, 1992*b*). This presents the possibility that some T cells may be autoreactive and, therefore, potentially harmful. In our pilot clinical study (Gjertsen *et al.*, 1996) this did not seem to be the case, since no side-effects or possible autoreactivity were observed in the patient having cross-reactive T cells. In a mouse model system, where $CD8^+$ T cells specific for different ras epitopes were induced following immunisation with a ras-vaccinia virus construct, only T cells specific for mutant ras were able to lyse target cells harbouring a *ras* mutation (Skipper *et al.*, 1993), indicating that endogenous expression of p21 ras by normal cells may only result in subthreshold amounts of ras peptide and is therefore insufficient for T-cell recognition. In this context, it is of importance that in many cases of human malignancies, the *ras* oncogene family seems to be overexpressed compared with normal tissues (Slamon *et al.*, 1984; Spandidos and Kerr, 1984). The basis for the cross-reactivity against the common mutations in position 12, as well as against the peptide expressing normal ras observed in donor 2, seemed to be a result of *in vivo* activation of a set of T cells, which was cross-reactive. The precursor frequency of these cells (1:110 000) was lower than the precursor frequency of T cells recognising the Asp12 mutation. This precursor frequency may be too low to cause any side-effects in the form of autoimmunity.

In conclusion, we have shown that ras peptide vaccination of patients with pancreatic adenocarcinoma can result in the induction of T cells specific for combinations of ras peptides and HLA class II molecules not previously demonstrated. These T cells are functionally active and can kill autologous peptide-pulsed target cells specifically. Such T cells may be of clinical benefit to patients with minimal residual disease. Studies are currently under way to test this approach in patients with colorectal adenocarcinoma and pancreatic adenocarcinoma after surgery.

**Acknowledgements**
This work was supported by the Norwegian Cancer Society. We thank Mrs G Jørum and Mrs K Lislerud for excellent technical assistance and Dr AV Reisaeter for helpful advice.

### References

ALMOGUERA C, SHIBATA D, FORRESTER K, MARTIN J, ARNHEIM N AND PERUCHO M. (1988). Most human carcinomas of the exocrine pancreas contain mutant c-K-*ras* genes. *Cell*, **53**, 549–554.

BOON T, CEROTTINI JC, VAN DEN EYNDE B, VAN DER BRUGGEN P AND VAN PEL A. (1994). Tumor antigens recognized by T lymphocytes. *Annu. Rev. Immunol.*, **12**, 337–365.

CAPELLA G, CRONAUER-MITRA S, PEINADO MA AND PERUCHO M. (1991). Frequency and spectrum of mutations at codons 12 and 13 of the c-K-*ras* gene in human tumors. *Environ. Health Perspectives*, **93**, 125–131.

DER CJ, FINKEL T AND COOPER GM. (1985). Biological and biochemical properties of human *ras* H genes mutated at codon 61. *Cell*, **44**, 167–176.

FOSSUM B, GEDDE-DAHL T III, HANSEN T, ERIKSEN JA, THORSBY E AND GAUDERNACK G. (1993). Overlapping epitopes encompassing a point mutation (12 Gly→Arg) in p21 ras can be recognized by HLA-DR, -DP and -DQ restricted T cells. *Eur. J. Immunol.*, **23**, 2687–2691.

FOSSUM B, GEDDE-DAHL T III, BREIVIK J, ERIKSEN JA, SPURKLAND A, THORSBY E AND GAUDERNACK G. (1994). p21-ras-peptide-specific T-cell responses in a patient with colorectal cancer. $CD4^+$ and $CD8^+$ T cells recognize a peptide corresponding to a common mutation (13Gly→Asp). *Int. J. Cancer*, **56**, 40–45.

FOSSUM B, OLSEN AC, THORSBY E AND GAUDERNACK G. (1995). $CD8^+$ T cells from a patient with colon carcinoma, specific for a mutant p21 ras derived peptide (13Gly→Asp), are cytotoxic towards a carcinoma cell line harbouring the same mutation. *Cancer Immunol. Immunother.*, **40**, 165–172.

GAUDERNACK G AND LUNDIN KEA. (1989). Rapid immunomagnetic phenotyping of cells. *J. Immunogenet.*, **16**, 169–175.

GEDDE-DAHL T III, SPURKLAND A, ERIKSEN JA, THORSBY E AND GAUDERNACK G. (1992*a*). Memory T cells of a patient with follicular thyroid carcinoma recognize peptides derived from mutated p21 ras (Gln→Leu61). *Int. J. Immunol.*, **4**, 1331–1337.

GEDDE-DAHL T III, ERIKSEN JA, THORBSY E AND GAUDERNACK G. (1992*b*). T-cell responses against products of oncogenes: generation and characterization of human T-cell clones specific for p21 ras-derived synthetic peptides. *Hum. Immunol.*, **33**, 266–274.

GEDDE-DAHL T III, FOSSUM B, ERIKSEN JA, THORSBY E AND GAUDERNACK G. (1993). T cell clones specific for p-21 ras-derived peptides: characterization of their fine specificity and HLA restriction. *Eur. J. Immunol.*, **23**, 754–760.

GEDDE-DAHL T III, SPURKLAND A, FOSSUM B, THORSBY E AND GAUDERNACK G. (1994). T-cell epitopes encompassing the mutational hot spot position 61 of p21 ras. Promiscuity in ras peptide binding to HLA. *Eur. J. Immunol.*, **24**, 410–414.

GILLIS S, FERM M, OU W AND SMITH KA. (1978). T-cell growth factor: parameters of production and a quantitative microassay for activity. *J. Immunol.*, **120**, 2027–2032.

GJERTSEN MK, BAKKA A, BREIVIK J, SAETERDAL I, SOLHEIM BG, SØREIDE O, THORSBY E AND GAUDERNACK G. (1995). Vaccination with mutant ras peptides and induction of T-cell responsiveness in pancreatic carcinoma patients carrying the corresponding RAS mutation. *Lancet*, **346**, 1399–1400.

GJERTSEN MK, BAKKA A, BREIVIK J, SAETERDAL I, GEDDE-DAHL III T, STOKKE KT, SOLHEIM BJ, EGGE TS, SØREIDE O, THORSBY E AND GAUDERNACK G. (1996). *Ex-vivo* ras peptide vaccination in patients with advanced pancreatic cancer: Results of a phase I/II study. *Int. J. Cancer*, **65**, 450–453.

JOHANSEN BH, GEDDE-DAHL T III, SOLLID LM, VARTDAL F, THORSBY E AND GAUDERNACK G. (1994). Binding of ras oncogene peptides to purified HLA-DQ (alpha 1*0102, beta 1*0602) molecules. *Scand. J. Immunol.*, **39**, 607–612.

JUNG S AND SCHLUESENER HJ. (1991). Human T lymphocytes recognize a peptide of single point-mutated, oncogene ras proteins. *J. Exp. Med.*, **73**, 273–276.

KAHN SM, JIANG W, CULBERTSON TA, WEINSTEIN B, WILLIAMS GM, TOMITA N AND RONAI Z. (1991). Rapid and sensitive non-radioactive detection of mutant K-*ras* genes via 'enriched' PCR amplification. *Oncogene*, **6**, 1079–1083.

MUKHERJI B, CHAKRABORTY NG, YAMASAKI S, OKINO T, YAMASE H, SPORN JR, KURTZMAN SK, ERGIN MT, OZOLS J, MEEHAN J AND MAURI F. (1995). Induction of antigen-specific cytolytic T cells *in situ* in human melanoma by immunization with synthetic peptide-pulsed autologous antigen presenting cells. *Proc. Natl Acad. Sci. USA*, **92**, 8078–8082.

OROSZ CG, ADAMS PW AND FERGUSON RM. (1987). Frequency of human alloantigen-reactive T lymphocytes. *Transplantation*, **43**, 718–724.

SEEBURGH PH, COLBY WW, CAPON DJ, GOEDDEL DV AND LEVINSON AD. (1984). Biological properties of human c-Ha-*ras*I genes mutated at codon 12. *Nature*, **312**, 71–75.

SKIPPER J AND STAUSS HJ. (1993). Identification of two cytotoxic T lymphocyte-recognized epitopes in the ras protein. *J. Exp. Med.*, **177**, 1493–1498.

SLAMON DJ, deKERNION JB, VERMA IM AND CLINE MJ. (1984). Expression of cellular oncogenes in human malignancies. *Science*, **224**, 256–262.

SPANDIDOS DA AND KERR IB. (1984). Elevated expression of the human *ras* oncogene family in premalignant and malignant tumours of the colorectum. *Br. J. Cancer*, **49**, 681–688.

TASWELL C. (1981). Limiting dilution assays for the determination of immunocompetent cell frequencies. I. Data analysis. *J. Immunol.*, **125**, 1614–1619.

TSANG KY, NIERODA CA, DE FILIPPI R, CHUNG YK, YAMAUE H, GREINER JW AND SHLOM J. (1994). Induction of human cytotoxic T cell lines directed against point-mutated p21 ras-derived synthetic peptides. *Vaccine Res.*, **3**, 183–193.